# Sclerostin as a biomarker of physical exercise in osteoporosis: A narrative review

**DOI:** 10.3389/fendo.2022.954895

**Published:** 2022-12-05

**Authors:** Anna Oniszczuk, Agnieszka Kaczmarek, Mateusz Kaczmarek, Maria Ciałowicz, Ersan Arslan, Ana Filipa Silva, Filipe Manuel Clemente, Eugenia Murawska-Ciałowicz

**Affiliations:** ^1^ Department of Physiology and Biochemistry, Wroclaw University of Health and Sport Sciences, Wroclaw, Poland; ^2^ Gynecology and Obstetrics Department, St. Hedwig’s of Silesia Hospital, Trzebnica, Poland; ^3^ Physiotherapy Faculty, Wroclaw University of Health and Sport Sciences, Wroclaw, Poland; ^4^ Faculty of Sport Science, Gaziosmanpaşa University, Tokat, Turkey; ^5^ Escola Superior Desporto e Lazer, Instituto Politécnico de Viana do Castelo, Rua Escola Industrial e Comercial de Nun’Álvares, Viana do Castelo, Portugal; ^6^ Research Center in Sports Performance, Recreation, Innovation and Technology (SPRINT), Melgaço, Portugal; ^7^ The Research Centre in Sports Sciences, Health Sciences and Human Development (CIDESD), Vila Real, Portugal

**Keywords:** sclerostin, osteoporosis, bone mineral density, physical activity, exercise, physical training

## Abstract

Osteoporosis, a disease of low bone mass, is characterized by reduced bone mineral density (BMD) through abnormalities in the microarchitecture of bone tissue. It affects both the social and economic areas, therefore it has been considered a lifestyle disease for many years. Bone tissue is a dynamic structure exhibiting sensitivity to various stimuli, including mechanical ones, which are a regulator of tissue sclerostin levels. Sclerostin is a protein involved in bone remodeling, showing an anti-anabolic effect on bone density. Moderate to vigorous physical activity inhibits secretion of this protein and promotes increased bone mineral density. Appropriate exercise has been shown to have an osteogenic effect. The effectiveness of osteogenic training depends on the type, intensity, regularity and frequency of exercise and the number of body parts involved. The greatest osteogenic activity is demonstrated by exercises affecting bone with high ground reaction forces (GRF) and high forces exerted by contracting muscles (JFR). The purpose of this study was to review the literature for the effects of various forms of exercise on sclerostin secretion.

## Introduction

Osteoporosis, as a disease of low bone mass, has been the subject of numerous studies worldwide for many years. The underlying cause of this disease is a disturbance of metabolic processes in bone tissue leading to excessive bone fragility (1). Recently, increasing scientific attention has focused on the protein called sclerostin, which, while influencing the balance between bone tissue formation and resorption, simultaneously exhibits sensitivity to mechanical stimuli (2). This fact became the basis for research on the effects of physical exercise on bone tissue metabolism, including the processes that cause osteoporosis (3–5). In the present study, the relationship between the physical activity and exercise level and the preservation or increase in bone mineral density was correlated with the level of sclerostin in bone tissue.

## Osteoporosis – low bone mass disease

Osteoporosis is a disease of the skeletal system characterized by increased bone fragility due to decreased bone mass and disruption of the microarchitecture of bone tissue. It is a disease that does not manifest obvious symptoms for a long time, despite its progressive, destructive effects on bone tissue. The first noticeable symptom is an osteoporotic fracture, otherwise known as a low-energy fracture (a spontaneous fracture caused by falling from one’s standing position height or minor trauma) (4). Osteoporosis is diagnosed when bone mineral density (BMD) reaches a value of less than 2 standard deviations, compared to the average BMD value in young people (6, 7).

## Osteoporosis as a lifestyle disease

Osteoporosis is recognized as a lifestyle disease on a global scale (8, 9). In 2010, 22 million women and 5.5 million men were diagnosed with low bone mass disease in European Union countries, while the number of new fractures was 3.5 million (7), 800,000 more have already been recorded in 2019 (6, 7). The most numerous fractures occurred in the proximal femur. In 2019, 25.5 million women and 6.5 million men were estimated to have osteoporosis in the European Union plus Switzerland and the United Kingdom. The population age 50 years or more is projected to increase by 11.4% in men and women between 2019 and 2034 and the annual number of osteoporotic fractures in those countries will increase by 25% (10). In Poland, 2 million patients over 50 years of age suffered from osteoporosis in a given year, and among them 168,000 suffered a fracture, 60% of them were women (7).

An osteoporotic fracture occurring as a result of decreased bone mass can lead to disability, especially in the case of femoral neck fractures. It devastates stabilization of life and leads to the reduction of its quality (11). Nowadays, 1 of 3 women over 50 years old (over breast cancer) and 1 of 5 men over 50 years old (over prostate cancer) are affected by osteoporosis (11, 12).

Osteoporosis is not only a social problem, but also an economic one. There is an increase in the aging population in developed countries. The economic burden of incident and prior fragility fractures in 2019 was estimated at € 57 billion in European Union countries with Switzerland and United Kingdom (13). The population of elderly people (aged 65 years or more) in European Union countries will increase significantly, rising from 90.5 million at the start of 2019 to reach 129.8 million by 2050. This age structure of population and increased percentage of elderly people will increase the prevalence of osteoporosis. Consequently, this will increase the monetary outlay associated with both the treatment immediately following the fracture and the lengthy rehabilitation and subsequent care. The cost of treatment is estimated to increase from 593 million euros in 2010 to 753 million euros in 2025 (14, 15).

## Causes and risk factors

The main cause of osteoporosis is low bone mineral mass, which depends on two types of factors: non-modifiable (impossible to eliminate) and modifiable (possible to change or eliminate).

*Non-modifiable factors:*

- age (there is a slow decline in bone mass after the age of 30);
- sex (women develop the disease four times more often than men);
- ethnic group (most common in Caucasian and Asian women);
- genetic conditions.

*Modifiable factors:*

- diet, eating habits (too little in the diet: vit. D, C and K, magnesium, phosphorus, potassium, omega 3 fatty acids, isoflavonoids; excess in diet: protein, vitamin A, sodium, alcohol, caffeine; smoking cigarettes);
- reduced physical activity;
- presence of other diseases (including hyperthyroidism, diseases affecting bone metabolism, diseases associated with impaired absorption, anorexia);
- use of certain medications (e.g., anticonvulsants, heparin, glucocorticoids) (14, 16, 17).

## Symptoms of osteoporosis

Osteoporosis is a disease that is asymptomatic, especially in its early stages. Very often, the first symptom of already advanced disease is the so-called osteoporotic fracture (or low-energy fracture) (18). These fractures usually involve the proximal end of the femur, the proximal end of the tibia, the spine, the pelvis, the proximal forearm, the proximal humerus and the ribs (19). According to Perry et al. (20), an osteoporotic fracture is a fracture that is disproportionate to the forces causing it, and occurs after a fall from one’s own standing height level, after ruling out another cause such as a pathological fracture. The risk of fracture doubles with a 10% decrease in BMD from the mean value (5). Low-energy fractures are followed by pain of varying degrees of intensity when performing simple motor activities, such as sitting down, bending the trunk, and even when standing. As the disease progresses, along with successive fractures, there is a limitation of mobility, a decrease in body height by about 2 – 4 cm, skeletal deformation, deepening of spinal kyphosis (the so-called widow’s hump), and symptoms of the respiratory, circulatory and digestive systems appear as a result (16).

According to many authors physical exercise ought to be one of the most suitable strategy in prophylaxis of osteoporosis, especially in postmenopausal women but not only, as a crucial element of life style (21–24).

## Sclerostin – bone remodeling protein

Sclerostin is a human bone tissue protein encoded by the SOST gene. It is located on chromosome 17 in the 17q12-q21 region (25). Sclerostin belongs to the bone morphogenetic protein (BMP) family of antagonists, and is involved in the anti-anabolic processes of bone formation (26). There are several regulatory elements in the SOST gene responsible for its transcription in bone tissue cells (27). Sclerostin was first detected in adult human osteocytes through the study by Winkler et al. (26). Studies have also shown the presence of this protein in hypertrophic chondrocytes (28). Sclerostin is a strong inhibitor of osteoblastogenesis (29, 30).

**Figure 1:**
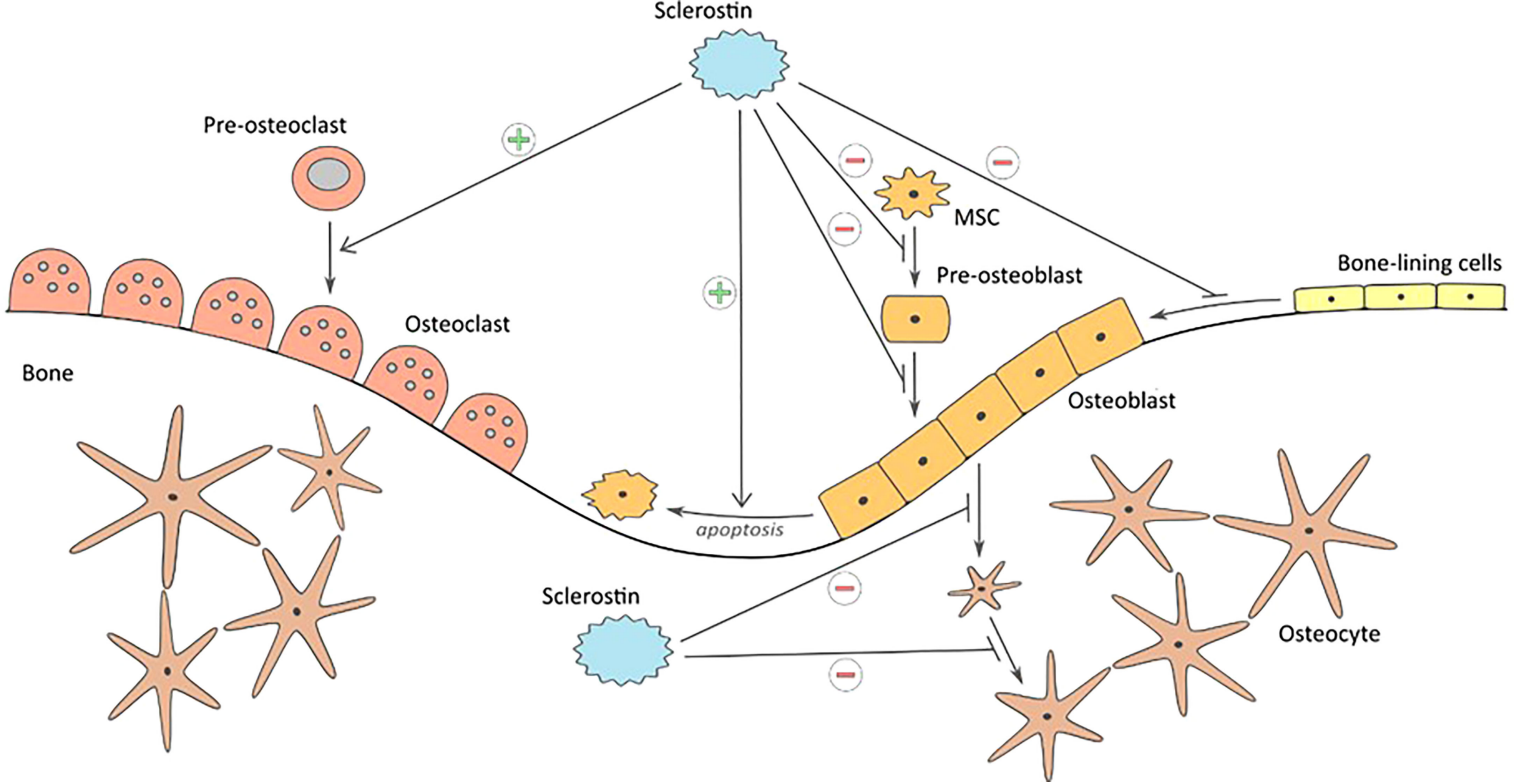
Influence of sclerostin on bone formation and resorption: inhibiting proliferation and differentiation of mesenchymal cells to osteoblasts, keeping the bone lining cells in dormant state, inhibition of bone matrix formation, inhibition of ostoblasts differentiation to osteocytes, promoting osteoblast apoptosis, and stimulating bone resorption.

This protein plays a key role in maintaining the balance between the processes of bone formation and resorption (bone remodeling) (**Figure 1**). It is a specific negative regulator of bone formation. Expression of this protein occurs in bone, cartilage, kidney, liver, pancreas and heart, among others, but it is mainly produced in bone tissue by mature osteocytes and cementocytes, and is detectable in plasma (31). Studies in genetically modified mice have shown that deletion of the SOST gene in the rodent genome resulted in high bone mass, a characteristic of humans with hereditary sclerostin deficiency (27). Sclerostin is released to inhibit bone formation. Its production is mainly regulated by mechanical loads on bone tissue and hormones affecting bone metabolism (calcitonin, parathyroid hormones, glucocorticoids). Calcitonin inhibits osteoclast resorption and up-regulates sclerostin expression by osteocytes. Glucocorticoids increase sclerostin expression *in vivo* and *in vitro* as well but there is a difference between results, probably due to different treatment regimens (32). Moreover studies have shown that serum sclerostin concentration in humans and expression in rodent bone tissue decreased in response to PTH treatment. Although sclerostin acts in a paracrine manner, changes in bone cell activities partly regulated by osteocytes may be reflected by circulation of sclerostin concentrations (33).

Mechanical stimuli damaging the bone tissue are perceived by osteocytes as changes in cytoplasmatic space. This leads to inhibiting the expression of sclerostin and to initiation of the bone tissue repair and formation processes (34). Exogenous sclerostin added to osteogenic cultures inhibits proliferation and differentiation of mouse and human osteoblastic cells. Moreover it decreases their life span by stimulating their apoptosis. Since sclerostin inhibits osteoblasts stimulation and bone formation processes, it leaves cells lining the bone tissue at rest (35). Moreover, studies have shown that another extracellular matrix protein – periostin - impacts on inhibition of sclerostin (36). The activity sclerostin as a regulator in bone tissue metabolism is dependent on the Wnt/β-catenin signaling pathway, whose modulator is periostin (37). The protein reacts directly with sclerostin and inhibits its antagonistic effect on this signaling pathway. As a consequence periostin promotes bone formation process. The study conducted by Bonnet et al. (38) has shown that periostin presence inhibits sclerostin expression and thereby increases level of osteoblasts. Mutual interaction of these two proteins has impact on bone tissue formation process in response to biomechanical loads.

## Sclerostin as an inhibitor of the Wnt/β pathway – catenin

**Figure 2:**
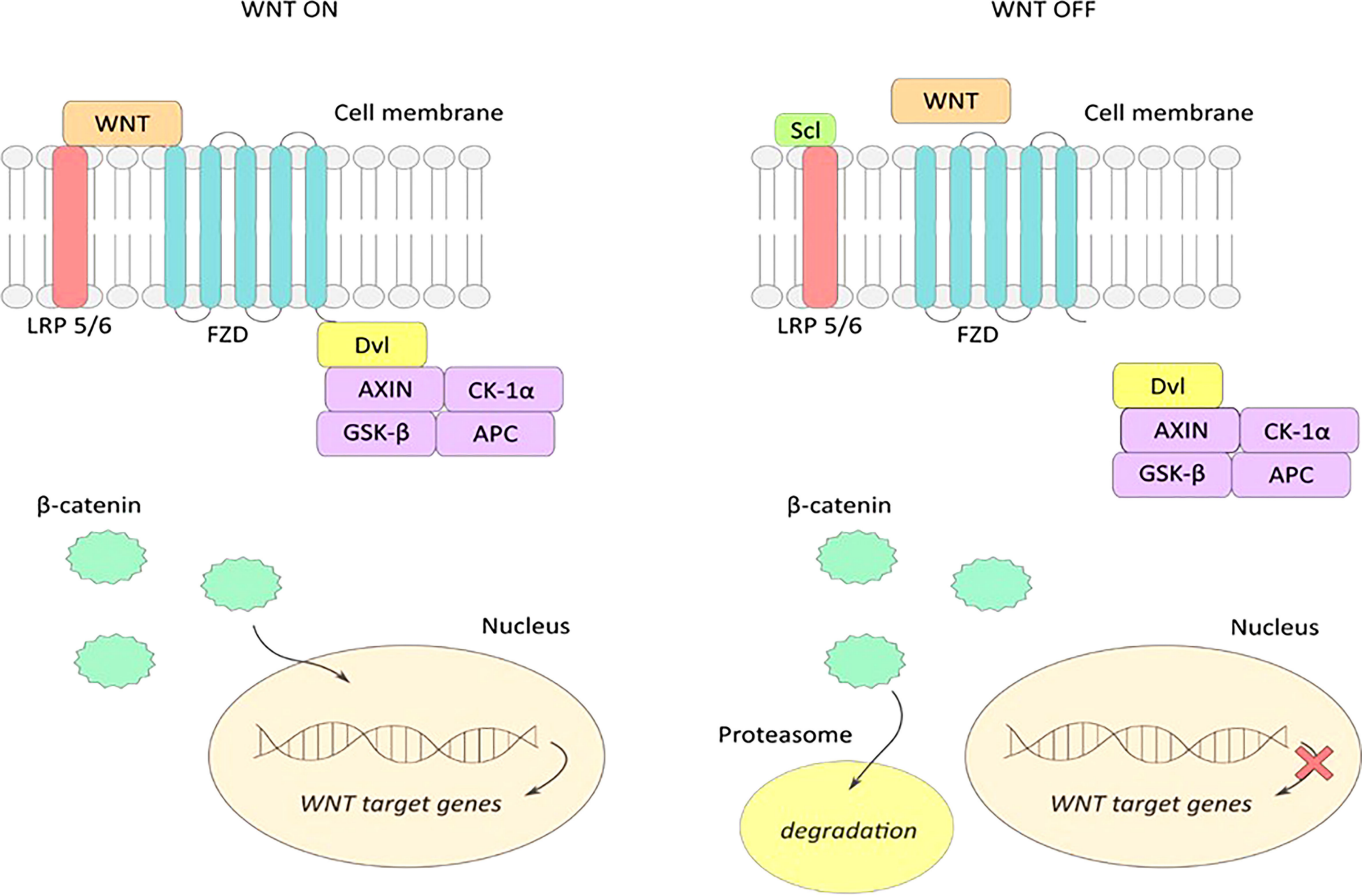
WNT ON-active signaling pathway: extracellular Wnt proteins bind to LRP5/6 and frizzled (FZD) receptors, and form an active Wnt-FZD-LRP5/6 receptor system leading to accumulation of the active form of β-catenin and its translocation to the cell nucleus. Attachment of β-catenin to the transcription factor TCF activates transcription of Wnt pathway target genes. WNT OFF – inactive signaling pathway: sclerostin binds to LRP5/6 receptors on the cell surface preventing the formation of an active Wnt-FZD-LRP5/6 complex, resulting in inhibition of the WNT signaling pathway. Accumulated β-catenin is degraded in the proteasome, and transcription of the WNT gene in the nucleus is stopped.

The Wnt pathway proteins form a ligand for Frizzled and lipoprotein receptor-related proteins (LPRs) located in the plasma membrane of the target cell. As low-density lipoproteins (LDL), LPR receptors have transport and signaling roles in the pathway (30). Once proteins bind to their receptors, the conduction of signals from the cell membrane to the cell nucleus is triggered, where gene expression occurs. The combination of Dvl (Dishevelled) protein with Frizzled receptor and axin with LRP receptor further leads to the activation of β-catenin, which then combines with TCF/LEF (T- cell transcription factor/lymphocyte enhancer factor) transcription factors to form an active complex leading to the expression of target genes. Lack of Wnt protein expression or inhibition of their attachment to receptors degrades β-catenin and inactivates the signaling pathway (39). As an inhibitor of the Wnt pathway, sclerostin binds to LRP5/6 receptors and masks them from Wnt proteins (27). This blocks the formation of the Wnt-Frizzled-LRP5/6 system leading to inactivation of signaling pathway transmitters. This ultimately leads to inhibition of anabolic processes of bone tissue by deactivating osteoblast differentiation (40) (**Figure 2**). Additionally, *via* the Wnt pathway, the lifespan of osteoblasts is prolonged by inhibiting their apoptosis (39).

The discovery of the effect of sclerostin on Wnt pathway signaling may be crucial in the prevention and treatment of bone remodeling disorders. Studies in mice and rats have shown that increased mechanical loading on bone tissue resulted in decreased sclerostin activity by osteocytes (41). Similar studies in wild-type mice have shown that mechanical stress relief of tissue has the effect of increasing sclerostin production, which in turn reduces the activity of the Wnt pathway (42). According to Sharif et al. (43) downregulation of sclerostin might be effective in the treatment of osteoporosis (44). conducted an experiment in which 7180 postmenopausal women suffering from osteoporosis were randomly divided into two groups – one group received romosozumab, a monoclonal antibody binding sclerostin, and the second group received placebo for 12 months. The risk of vertebral fractures in women receiving romosozumab was 73% lower, compared to placebo group. Also according to Brandenburg et al. (45) blocking sclerostin is a quite promising treatment perspective against osteoporosis moreover authors underly the Wnt signaling pathway and sclerostin secretion with evident cardiovascular calcification observed in different diseases.

## Effect of physical activity on sclerostin and bone mass

The precise influence of physical training on sclerostin level stays unclear. Many studies show a negative correlation between increased physical activity and sclerostin level. Ardawi et al. (32, 46) conducted two experiments including premenopausal women divided into two groups, one of which consisted of physically active women, and the second one - sedentary women. In both experiments, blood and urine levels of sclerostin were significantly lower in physically active women. Similar results were obtained in women aged 50-75, suffering from osteopenia, by Janik et al. (47). Exposing osteocytes to sera of obese women undergoing physical activity program shows negative correlation between duration of the program (sera were collected 48 hours before training program, and then after 4, 6 and 12 months of training) and sclerostin level (48). Similar results were achieved by Wannenes et al. (49), who also noted lower mRNA levels of some key osteogenic factors, like Runx2, BNP4 and BALP, compared to control group. There was also a significant decrease in expression of cMyc and axin2, specific target genes of canonical Wnt/β-catenin signaling pathway.

Studies including male participants show corresponding results. Hinton et al. (50) conducted a study in apparently healthy men aged 25 to 60 years whose physical activity in the past 24 months was ≥4 hours per week. The study group was divided into those doing resistance training or jump training and underwent their 12 month intervention. After this time, a significant decrease in serum sclerostin levels was examined and observed.

However, there are many experiments showing results contrary to the above. Pickering et al. (51) subjected young, healthy women to a 45-minute treadmill run. They achieved a significant increase in sclerostin level. Similar results were obtained by Gombos et al. (52), who observed an increase in sclerostin level after a single exercise session in both the resistance exercise and walking groups, compared to its baseline level.

Kouvelioti et al. (53) studied young, healthy women and subjected them to two exercise tests: interval running on a treadmill and cycling on a cycle ergometer. They obtained an increase in sclerostin level after training in both trials. Interestingly, sclerostin levels returned to baseline values one hour after the end of training regardless of the exercise regimen.

During a study conducted by Armamento-Villareal et al. (54) older, obese individuals were randomly assigned to a control group that included diet or exercise, and exercise combined with diet. Attempts were made to see how weight loss would affect serum sclerostin levels. After a 12-month study, there was an increase in sclerostin in the diet group. It remained unchanged in the other groups. Śliwicka et al. (55) conducted a study in healthy men after a marathon. Sclerostin levels were observed to increase 1.3-fold 72 hours after the marathon compared to baseline.

Detailed information of different studies about influence of various form of physical activity/training in healthy/obese/athletes are presented in **Tables 1**, **2**.

**Table 1:** Studies showing the effect of physical activity on changing sclerostin levels.

Ref:	Group	GroupCharacteristic	Type of physical activity/ /exercise/ training	Sclerostin	Other biochemicalparameters	Additional effects/Comments
(32)	♀ n=120	Age: 30-42 years Premenopausal;BMI: 30.0 kg/m^2^ or less; sedentary lifestyle; stable body mass; not being on the special diet; lack of participation in another program during the study; randomly classified to PA training group (PAT) or sedentary (SG);	Duration: 8-week 120 min/ session, 4d/wk; (20 min walking, 25 min running, 10 min cycling, 10 min step ups, 35 min stretching and mobilizing the spine, upper and lower body);	↓ Sclerostin level by 33.9% ( 26.06 pmol/L pre-test and 19.46 pmol/L post-test) in PAT group; CG: no changes 25.69 pmol/L before, 26,41 pmol/L post- test;	PAT : ↑ IGF-1 pre- 50.26 ng/ml to 87.54ng/ml;↑ BALP pre 8.16 U/L to 12.01 U/L after test; ↓CTX form 166.5 to 151.5pg/ml;↑ intact parathormone (PTH) from 2.76pmol/L to 3.38 pmol/L;	Exclusion criteria as in ref (46).;No correlation were observed between Sclerostin and bone resorption markers in PAT group;
(32)	♀ n=1235	Age: 33.83 ± 8.41yearsHealthy; Premenopausal; Serum FSH level ≤15mIU/L and a normal cycle; normal blood count, renal and hepatic tests; All inclusion data as above;	All group divided into four groups based on PA level: <30, 30-60, 60-120, >120 (min/week);	↓Sclerostin level by 36% ( 17.60 pmol/L) in the groupwith PA >120 min/wk compared to the PA < 30 min/wk. (27.84 pmol/L). Sclerostin level in group with PA = 30-60 min/wk =27.11 pmol/L, and in group with PA = 60-120 min/wk Sclerostin level =21.64 pmol/L;	IGF was the highest in PA group >120min/wk (101.89ng/ml) and the lowest in PA group <30 min/ wk (49.27ng/ml);BALP was the highest in PA group >120min/wk (11.13U/L) and the lowest in PA group < 30min/wk (8.93U/L);CXT was also the highest in PA group >120min/wk (238.5pg/ml) and the lowest in PA group <30 min/ wk (191.7 pg/ml);	Exclusion criteria the sameas ref (46).;No correlation were observed between Sclerostin and bone resorption markers in PAT group;
(41)	♂ n=8	Age: 25.0 ± 4.0 years Range: 18-30 years; Obese; exercised no more 2-3x /week (150 min); waist circumference > 98cm; no cardiometabolic diseases, no medication, non-smoking; BMI = 35 ± 4 kg/m^2^;	Duration: 4 weeks of sprint interval training (SIT); 4 session/week /4 weeks on cycle ergometer; Session: 5 min warm-up, 8 x 20s work at 170% work rate at VO_2peak_/ 10s rest, total time =9 min; Post training serum and subcutaneous white adipose tissue (WAT) biopsy have been taken;	↓Sclerostin in serum 15 % pre- to post- SIT, 5/7 showed decrease, n.s.);WAT - ↓sclerostin (37% pre v post);	↑Wnt/ β-catenin signaling in WAT (52%); ↓ TNF-α (−0.36 pg/ml) and IL-6 (−1.44 pg/ml);	VO_2 peak_ increased (5%); no anthropometric changes after 4 weeks; Sclerostin in regulating human adipose tissue in response to exercise training;
(46)	Women (♀) n=50	Age: 64.8 ± 5.0years; Range: 50-75 years;With clinically diagnosed osteopenia;	Duration: 12-week observation /12-weeks physical activity; Interval training on a cycle ergometer 4 min exercise/2 min rest, 3 times a week for 40 min);	↓ Sclerostin 12.04% (275.82 ng/mL pre-test and 242.60 ng/mL post-test);	↑ Osteocalcin (OC) level from 21.67 ng/mL to 23.64 ng/mL after the study; ↑ vit.D_3_ from 23.7 ng/ml to 32.55 after study; no changes of C-terminal telopeptide type 1 collagen (β-CTX/ β-CrossLaps); no changes of Alkaline Phosphatase (ALP) activity, Phosphorus and Calcium (Ca) level;	Supplementation with vit. D_3_ (1800IU) and Ca (500mg) during entire study in all women. No significant correlations between OC and Sclerostin;
(50)	♀ n=28	Age - 53 ± 8.2 years Obese; BMI≥ 30 kg/m^2^; Body mass; 101.3±3.9 kg; Sedentary lifesyle;	Duration: 12–months; daily aerobic training; individualized prescribe physical activity and hypo caloric diet.Time of training session varied from 30 min/2 months and 60 min to the end of study;	↓ Sclerostin levels after 4, 6 and 12 months compared to baseline;	Decrease of insulin and leptin levels; increased of adiponectin receptor-1 (Adipo R1) after 6 and 12 months; time-dependent total β-catenin increase and others intracellular markers;	Significant reduction of body mass (to 91.0 ± 9.5 kg after 12 months due to fat and fat free mass; Body composition variation achieved after 4 months and maintained for for the end of study;
(52)	Men (♂) n=38	Age: 43.7 ± 10. 1years Range: 25-60 years. Healthy, physically active (≥4 hours of leisure-time physical activity/week with low lumbar spine or hip BMD (>-2.5 SD T-score ≤ -1.0 SD);	Duriation: 12 months; All group randomized into two groups: (RT) resistance training and (JUMP) jump training; 8 cycles of 6 weeks training/1 week rest, progressive intensity; JUMP – 3x/wk; RT 2x/wk;	↓ Sclerostin levels by about 7% from 39.2± 11.6 pmol/L to 36.8± 13.3 pmol/L in both group;Mean % of change was −4.5 ± 3.6% for JUMP and −9.5 ± 3.5% for RT;	IGF-I increase of 26% from 203± 71ng/mL to 239± 109ng/mL in both group;PTH - no changes; Whole body and LS BMD increased after 6 months in both groups; Hip BMD significantly increased at 6 and 12 months only in RT;	All participants were provided supplementation with Ca (1200mg calcium carbonate/d) and vit. D (10 μg/d);
(53)	♀ n=32	Healthy, Two groups:Practicing PA less than 120min/wk (age: 22.9± 1.5years) n=23; Resting test (age: 26.1±3.1 years) n=9;	Duration: 45 min low-speed, treadmill running test;	↑Sclerostin levels in practicing PA group by 44.3% from 290 ± 19 pg/mL before test to 410± 27pg/mL after Resting test: Stabile level (303 vs 294 pg/ml);	Increase in level by 7.7%from 370.9+/-31.5 to 386.2+/-28.5 pg/mL);	
(54)	♀ n=150	Age: 58.80±7.5 years;With diagnosed osteoporosis/ osteopenia. Randomly assigned to three groups:Resistance (RG), Walking (WG), Control (CG);	Duration: 46 minRG: 8min warm-up, 30 min exercises with elements of core stabilization and muscle strengthening 3 sets/ 2 min rest, 8 min cool-down;WG: 46 min outdoor walking (3–6 MET), rhythm 100 steps/min CG: any intervention;	↑ Sclerostin levels in RG: pre - 6.8 pmol/L to 29.8 pmol/L post intervention; WG: pre- 23.6 pmol/L to 29.9 pmol/L post-; CG: Pre - 24.0 pmol/L v 24.20 pmol/L post intervention;	RG:↑CTX/β-CrossLaps) (303.60 to 276.40 pg/mL post intervention) WG:↑Bone-Specific Alkaline Phosphatase (BALP);	Exclusion criteria:Any condition influencing Ca and bone metabolism (expect dietary Ca and vit. D supplementation), Ongoing hormone replacement therapy, renal and hepatic diseases, cardiovascular disease, physical injury, anabolic steroids, anticoagulants, diuretics within last 6 months;
(55)	♀ n=20	Age: 22.5±2.7 years Range: 18-28 years. Healthy, recreationally active (2 to 5x/wk , free of injuries or chronic conditions, having no fracture in the last year, nonsmokers, and not taking any medication or dietary supplements (protein, vit. D, and calcium);	Duration: 16 min Two exercise tests:High intensity interval running (HIIR) on a treadmill and HII cycling on a cycle ergometer (HIIC);HIIR and HIIC lasted 8 x 1 min running /cycling at ≥90% of HR_max_ separated by 1 min passive recovery between work; During both trials 5x blood samples were collected: pre and 5 min, 1h, 24h, and 48h post exercise;	↑Sclerostin level in 5 min after exercise in both trials, in HIIR from 100.2 to 131.6 pg/mL and from 102.3 to 135.8 pg/mL in HIIC.Recorded significant effect of time but not exercise mode; at 1h after exercise Sclerostin level returned to pre- test value;	No significant time effect for CTXI in both trials; A significant time effect for procollagen type I amino-terminal propeptide (PINP) was found only in HIIR; No significant differences in CTXI and PINP concentrations between both trials at any time point. No significant correlations were found between the sclerostin, CTXI and PINP levels at any time point;	During both training mean heart rate was >90% of HRmax (93.2±4.7% for HIIR and 90.2±4.8% for HIIC) Borg rating of perceived exertion (RPE) was recorded in both trials = 19;
(56)	♀♂ n=107	Age: ≥65 years, Obese (BMI ≥30kg/m^2^); no physicaly active; stable body weight (±2 kg) in past year; on stable medications within last 6 months;	Duration: 12 weeks;All participants divided into four groups: control group, with diet induced weight loss, exercise training group, diet and exercise group. Exercised groups: 90 min (15 min flexibility exercise, 30 min aerobic, 30 min progressive resistant exercises 15 min balance exercise);	↑ Sclerostin levels in the diet group by 6.6± 1.7% and 10.5% ± 1.9% in 6 and 12 month compared to baseline. There was no changes in the other groups;	Body weight decreased in diet and in diet + exercise but not in exercise and control;	All received with Ca (1500 mg/d) and vit. D (1000 IU/day); Exclusion of subjects taking bone-acting drugs, sex-steroid compounds within last year;
(53)	♂ n=14	Age: 22.1 ± 4.05 years; Range: 18-39 years; Volunteers; Healthy, Active Duty Army Solders; not having used glucocorticoids in the past 2 years; BMI: 27.3±3.8 kg/m^2^	Single bout of exercise; Randomized crossover study; 10 sets /10 repetitions of plyometric jumps at 40% of 1 -RM leg press or a nonexercised control period; Blood was drawn at baseline, 12, 24, 48, 72h following exercise or rest	No significant effect of time or exercise on sclerostin levels;	Markers of bone metabolism: (PTH, Ca); markers od bone formation: bone Alkaline Phosphatase (BAP); osteocalcin (OCN); markers of bone resorption ( CXT (lower in 12h in comparison to baseline), Dickkpopf-1 (DKK-1);	Calcium controlled diet (1000mg/day) was implemented;
(57)	Girls and boys ♀ n=12 ♂ n=12	Age: ♀ - 11.00 ± 0.5 years, ♂ - 10.2 ± 0.3 years. All girls premenarcheal; all children recreationally active; no difference between ♀ and ♂ in daily energy intake and Ca intake, but below recommendation for children in this year. BMI <85^th^ percentile for their age; no fracture (within 6 past months); growth no premature or delayed, no pharmaceuticals;	Duration and exercise: High impact of plyometric exercise protocol in form of circuit training stations (3x): 5 min warm-up, different stations, 3 min rest between stations; exercises: jumping jacks, lunge jumps, single-leg hops, hurdle jumps, tuck jumps, drop jumps (entire session about 100-144 jumps);	↑Sclerostin in ♀ in comparison to boys before (♀-187.1 pg/ml v ♂-161.4 pg/ml) and at 24h post exercise (♀-200.3 pg/ml v ♂-162.9 pg/ml); In girls post exercise the level was lower in comparison to the pre exercise at 5 min and 1h, at 24h much higher than in previous stages. No changes in boys post exercise;	DKK-1 – ↓in ♀than in ♂ at the same time; no changes post exercise in both groups.OPG - ↓ in ♀ than in ♂ at the same time, except 24h;RANKL (receptor activator of nuclear factor kappa-β ligand) ↓ in ♀ than in ♂ in each stage of study; In ♀ post exercise lower then pre exercise; no changes in ♂ post exercise;	Plyometric training induces osteokine response favoring osteoblastogenesis than osteoclastogenesis;

♀, women, ♂ men, ↓ reduced level, ↑ decreased level.

**Table 2:** Studies depicting the effects of physical activity on the bone mass of professional athletes.

Ref:	Group	GroupCharacteristic	Type of physical activity/ training	Sclerostin	Other biochemicalparameters	Additional effects/Comments
(58)	♂ n=10	Age: 41±7.7 years Range: 32-51 years Healthy; recreational runners;	Visegrad Marathon (42.195 km);	↑ Sclerostin levels 1.3-fold 72 h after the marathon in comparison to the baseline;	24 h after marathon, an increase in myostatin (1.2-fold), osteoprotegerin (OPG) (1.5-fold) and PTH (1.3-fold), high-sensitive interleukin-6 (hsIL-6) (1.9-fold), myoglobin (4.1-fold), hs C-reactive protein (hsCRP) (5-fold), and tumor necrosis factor α (TNFα) (2.6-fold); After 72h and in myostatin (1.2-fold), irisin (1.1-fold). OPG (1.3-fold) and PTH(1.4-fold), hsIL-6 (1.4-fold), TNFα (1.9-fold);	Sclerostin was correlated with hsIL-6; negative correlation was noted fo sclerostin and myostatin and PTH and OPG;
(59)	♂ n=59	Range: 17-37 years; Healthy;Athletes-footballers (A) n=43; aged 26.5 ± 3.4 years body mass 76.3 ± 7.3 kg, BMI 23.1 ± 1.5kg/m^2^; Mean career duration 14.7 ± 4.5 years; Non-athletes (NA) n=16; Aged 29.5 ± 4.3 years, non-smokers; low physical activity per week; body mass 81.7±8.7 kg; BMI 25.6±3.1 kg/m^2^; All NA participants worked indoors;	Winter season; Training lasted every day by 3 h/d in uniforms covered 80% of their body;	↑ sclerostin in A group (35.3±8.9 pmol/L) than in the NA group (28.0 ± 5.6 pmol/L);	A group had higher concentrations of P1NP (145.6 ±77.5 vs 61.2 ± 22.3 ng/ml; and vit. D_3_ (16.9±8.4 vs 10.3 ± 4.3 ng/ ml; lower concentrations of PTH (25.8 ± 8.3 vs 38.2 ± 11.5 pg/ml in comparison to NA. VO_2_max = 56.09 ± 4.29 ml/kg/min in A group;	Vitamin D deficiency was found in 77% of A and 100% of NA;
(60)	♂ n=9	Age 28.8 +/- 3.6 years;Healthy, cyclists;	The 3-week stage cycling race Giro d’Italia 2012Saliva was collected at days:-1, 4, 8, 12, 14, 19, 23; Blood and urine were collected at days: -1, 1, 23;	↑Sclerostin; average level of sclerostin on the 1^st^ day: 254.5±134 pg/ mL, in 12^th^ day: 477.5± 137.9 pg/mL, in 23th day: 762.1 ± 143.3 pg/mL;	Cortisol remained constant, testosterone decreased at day 4, estradiol and DHEA firstly increased and then returned to basal levels. LDH, CK, AST, and urinary Ca and phosphorous increased;	DHEA and estradiol correlated with the physical effort and the bone-muscular markers;
(61)	♀ n=62	Age - 14-18 years;Eumenorrheic adolescents;Healthy;	Three study groups: rhythmic gymnasts (RG), swimmers (SW), untrained control group (UC);	↑ Sclerostin levels was higher in RG: (129.35 ± 51.01 pg/ml; by 74%) and SW; (118.05 ± 40.05 pg/ml; by 59%) v UC: (74.32± 45.41 pg/ml);	No differences between groups in preadipocyte factor-1 (Pref-1), Osteocalcin and CTx;	Adolescent have higher sclerostin compared to UC; Sclerostin correlated with whole-body BMD and lumbar spine (LS) areal bone mineral density (aBMD) in RG, and femoral neck aBMD in UC. No correlation was found between sclerostin and BMD in SW;
(61)	♂ , ♀ n=61 Control n=16 8 ♂ 8♀	Age: 27.2 ± 6.8 years; 15 – Italian national rugby team (29.1 ± 1.7 ys; 13 professional cycling team (31.1. ± 2.7 years); 6 professional tennis players (23.2 ± 6.2 years); 11 professional endure motorcycling team (29.1 ± 11.8 years); 8♀ professional basketball players firs Italian league (27.0 ± 3.0 s); 8♀ figure ice skaters Italian national (19.5 ± 4.9 years);	All athletes classified into three group based on work-load: - (1) weigh bearing, (WB: rugby, endure, basket), (2) non-weight bearing (NWB: cycling), (3) high impact sports (HI: ice skating, tennis); Blood taken after 10 min resting;	Sclerostin level was the same for entire group of athletes and control (0.42 ± 0.09 ng/ml, n.s.); Significant differences between genders in whole cohort: ♂-0.45 ± 0.07 ng/ml, ♀-0.40 ± 0.09 ng/ml) and sedentary group: ♂-0.36 ± 0.05 ng/ml, ♀-0.46 ± 0.09 ng/ml; Differences between men in athletes – rugby players (0.44 ± 0.11ng/ml) and endure (0.42 ± 0.04 ng/ml) had much higher Sclerostin level than cyclists (0.34 ± 0.08 ng/ml);	ALP – 22.4 ± 7.6U/L in athletes and 24.3 ± 8.5U/L in sedentary; Differences of ALP between whole cohort of men and women (21.3 ± 6.8 U/L v 26.1 ± 8.8 U/L) and in athletes: men (20.4 ± 5.5U/l v women (28.4 ± 9.8U/L); No differences in sedentary group. No differences in athletes men and women between sport categories;	Significant correlation were noted for sclerostin level and age; no differences within gender in entire athletes group. No correlation between sclerostin level and category of sport in females. No gender differences in athletes group (♂-0.41 ± 0.09 ng/ml v ♀-045. ± 0.07 ng/ml); No differences in ♀ group of athletes within sport category and to sedentary; In WB athletes sclerostin much higher (0.43 ± 0.0ng/ml) than in NWB athletes (0.34 ± 0.08 ng/ml);
(62)	♀ n=64	Age: 9-10 years Healthy; Gymnasts (RG), n=32; Untrained control (UC), n=32;	Comparison between two groups;	RG: Sclerostin 19.8 ± 6.3 pmol/l was higher in comparison to UC;	RG: Pref-1 (1.6±1.0 ng/ml) was higher than in control (untrained);	Sclerostin and Pref-1 levels are higher in RG compared to UC girls. Sclerostin was related to adiponectin in UC;
(63)	♂ n=9	Age: median 45 years; No specific inclusion and exclusion criteria; Healthy; amount of training about 100km during winter time and more than 200km during summer, up to 7000km/ year;	Spartathlon race 246 km (ultramarathon food race). Runners start in Athens and have to reach Sparta with 36h; It took them 34h 3 min (32h 29 min; 35h 3 min) to reach Sparta;	↓Sclerostin after the race (pre- 29.15 pmol/L v 27.75 pmol/L, post- race (n.s.);	Significant ↑myostatin (23.73 ng/ml v 26.73 ng/ml); ↑↑Follistatin (300.8 pg/ml v 1211 pg/ml; ↓ Dkk-1 (38,68 pmol/L v 38.14 pmol/L); ↓ P1NP (54.37 ng/ml v 41.14 ng/ml); ↑ CTX (0.299 ng/ml v 0.542 ng/ml); The increase of myostatin can reflect muscle catabolism processes induced by overstrenuous exercise;

♀, women, ♂ men, ↓ reduced level, ↑ decreased level.

Next to sclerostin there are some other bone formation and resorption biomarkers which can be considered in relation to physical effort. Studies conducted by Kouvelioti et al. have shown that sclerostin level increase after five minutes in response to high intensity exercises but PINP (procollagen type I amino-terminal propeptide) and CTXI (cross linkedtelopeptide of type I collagen) do not correspond to the sclerostin response. Moreover, no correlation between sclerostin and PINP or CTXI values at any time of exercises was noted (56). Gombos et al. conducted experiments on three groups: resistance exercise group, walking group and control group. Increase in sclerostin level in both study groups with significant difference was observed but there was no significant change in BALP values in any of the groups. Next, the changes in CTX concentrations were significant in the resistance exercise group but not in the walking group. Physical effort of appropriate type, intensity and duration may affect bone formation and resorption causing detectable changes in serum concentrations of biochemical markers of bone metabolism such as PINP, CTXI, BALP and sclerostin. Forces generated by muscle contraction play an important role in stimulating bone resorption (58).

## Physical activity of professional athletes and sclerostin level in bone tissue

Previous studies on the effects of physical activity levels on bone tissue sclerostin levels have shown that mechanical loading of bone tissue increases bone density, promotes tissue formation processes, and inhibits resorption. Are sclerostin levels at similar levels at very high exercise loads in professional athletes whose bones are subjected to daily high mechanical loads?

Many studies seem to support that thesis. Zagrodna et al. (53) compared sclerostin levels in professional football players and in healthy individuals with low levels of physical activity. A significantly higher mean level of sclerostin was observed in the football players group compared to the control group. Similar conclusions may be drawn from comparing sclerostin levels in athletes from many other sports with different workloads to people who do not practice any sports (60, 64).

Sclerostin levels, already high in professional athletes before physical effort, seem to grow even higher during long-term exercise. The study conducted by Grasso et al. (59) involved 9 professional cyclists who raced a total distance of 3503.8 km during the 3-week stage cycling race Giro d’Italia 2012. One of the many parameters measured was the mean sclerostin level in the blood samples of the competitors taken in the morning during the intervals between successive stages. The authors showed that the blood sclerostin level in the cyclists increases during the race in successive stages. The implication is that prolonged high-intensity exercise, as during a 3-week cycling race, may lead to increased bone resorption by steadily increasing serum sclerostin levels during exercise and maintaining high levels between activity stages. This wouldn’t be surprising, since there’s already data showing that consistent high loads due to continuous training stimulus increase the sclerostin level through increased bone metabolism (60), which is especially evident in strength sports (61).

## Physical training in the prevention of osteoporosis

Physical training to prevent bone mass loss and to maintain or increase BMD levels is based on different principles than training to improve cardiovascular or muscular capacity. When properly selected, composed and conducted, the training has an osteogenic effect, while improper training can lead to the so-called saturation of the osteogenic response to a mechanical stimulus. The bone tissue then becomes resistant to the training stimulus (5).

Exercise as a mechanical stimulus to the skeletal system increases bone mineral density through a mechanotransduction mechanism in bone, involving the sclerostin protein as described previously (62, 65). Based on that, the effectiveness of physical training in the prevention of skeletal disorders can be assessed by BMD, depending on factors such as:

- type of training (66);
- exercise intensity (63, 67);
- frequency of exercises, breaks between exercises and series (63, 67)
- the number of body parts involved (68)
- systematic approach (69)

Exercise to prevent osteoporosis must be of such intensity that bone tissue shows a threshold sensitivity to mechanical stimulus, because bones show an osteogenic effect only when this threshold is exceeded (70). Studies among menopausal women have confirmed the effect of high-intensity walking on increasing BDM, particularly in the lower body. The threshold for osteogenic activity in the study group occurred at a speed of just over 6.14 km/h and a load of 872.3 N, which translated to 80% of age-specific maximum heart rate (HRmax), 74% of VO_2_max, and 115% of ventilation threshold (71). If the stimulus intensity is increased during training or a training cycle, the potentiation of the osteogenic effect will occur until the so-called saturation of the osteogenic response (72).

As per Bailey et al. (69) daily exercises results in greater osteogenic activity. Moreover, Ardawi et al. (32) showed that physical activity levels above 120 min per week result in significantly higher serum sclerostin levels, leading to increased bone mineral density. Exercise should involve as many body parts as possible because of the fact that osteogenic activity occurs in the part of skeleton directly loaded by the mechanical stimulus (68). Breaks between repetitions of a given exercise in a cycle allow the mechanical stimulus to activates more bone-forming cells or osteoblasts and achieve an osteogenic effect with fewer repetitions, but also to shift the threshold at which saturation of the osteogenic response occurs later (73, 74). Moreover exercise should be repeated several to a dozen times, and the intervals between exercise cycles should be more than 4 - 8 hours in order to avoid saturation of the osteogenic response (72, 73).

Research to date confirms that exercise has a beneficial effect on bone health (75). However, the size of osteogenic effect obtained depends not only on the factors mentioned above, but also on the type of physical training performed (5). Exercise exerts two types of mechanical load on the bone in the form of JFR e.g. running, walking, climbing stairs and GRF e.g. rowing, weightlifting. A study of 39 postmenopausal women found that both types of exercise resulted in a significant increase in BDM, but GRF-based training resulted in a greater increase in both the entire body, and the individual skeletal parts tested (76). **Table 3** lists the types of exercise along with the degree of osteogenic effects (77).

**Table 3:** Types and examples of exercises with their corresponding osteogenic effect coefficient.

Type of physical exercise	Example	Osteogenic effect coefficient
Exercises without or with smallGRF and JRF	cycling, swimming	0
Exercises or games with small GRF and JRF	bowling, walking	1
Exercises or games with moderate GRF and JRF	dancing, aerobic exercises with light loads rhythmics	2
Exercises or games with GRF > 1000μE	Running, aerobic exercise with heavy loads,tennis, squash	3
Exercises with large JRF	strength training using equipment	3

High- and moderate-intensity exercise involving both JFR and GRF causes a strong osteogenic effect. The greatest osteogenic activity is found in running, tennis, and weight training using equipment, among others. In addition, a slightly higher mean BDM (across skeletal parts) was observed among women performing GFR-based exercise training (76). Power training based on dynamic exercises will be more effective in preventing osteoporosis than training based on strength exercises (72).

There is also an interesting question of the influence of the level of physical activity during childhood and adolescence on bone mass in elderly people. There’s data showing that peripubertal exercise causes at least two types of skeletal adaptations: periosteal expansion and better trabecular microarchitecture (78). Especially high sensitivity of the skeleton to exercise at this time of life may be due to high growth hormone level. The extent to which the forementioned skeletal changes may last to the old age remains unclear. Nevertheless, it is worth mentioning that structural changes may persist despite the loss of bone mass (79, 80)

Studies have shown that exercise programs which includes at least two kinds of activities such as weight-bearing activities, progressive resistance training (PRT) and/or power training and balance/mobility training have positive effect on skeletal system and fall-related risk factors (81). Detailed training program recommended in osteoporosis and osteoporotic fractures prevention with physical activities, frequency, intensity and sets/repetitions descriptions is presented in **Table 4**.

**Table 4:** Training program recommended in osteoporosis and osteoporotic fractures prevention (82).

Type	Progressive resistance training	Weight-bearing impact exercise	Challenging balance, steppingand mobility
**Exercises**	Exercises: squats, lunges, hip abduction/adduction, leg press, thoracic/lumbar extension, plantar/dorsi-flexion, abdominal/postural exercises, bent over row, wall/counter/floor pushup, triceps dips and lateral shoulder raises.	Multidirectional and novel loading activities: jumping, bounding, skipping, hopping, bench stepping and drop jumps or participation in weight-bearing sports (e.g., tennis, dancing, netball, recreational gymnastics and football).	Include static and dynamic movements: reduce base of support, shift weight to limits of stability (e.g., leaning/ reaching), perturb center of mass, stepping over obstacles, alter surface (foam mats) and multi-sensory activities (e.g. reduce vision) and dual tasking. Consider Tai Chi and rapid stepping movements in different directions.
**Frequency**	≥2 days per week	4- 7 times per week	Accumulate at least 2- 3 h per week. This could be achieved within other exercise bouts during the course of a week.
**Intensity**	Start with slow and controlled movements and emphasize correct lifting technique.Progress to 75-85% of 1-RM(5-7/8 on Borg 0-10 point RPE scale or hard-very hard).Consider progressing to high velocity (power) resistance and functional training for lower extremities to increase rate of loading and improve movement speed and power.Light-to-moderate loads (30-70% 1-RM) can be used.	Moderate to high impact activities (>2-4 BW), as tolerated.Increase height of jump, step heigh, weights or a weighted vest and incorporate change of direction movement.For sedentary individual and those with poor muscle strength or function, start with PRT for 6-12 weeks to strengthen lower limb muscles and/or introduce low impact exercises and core muscle training.	Must be progressively challenging (close to limit of balance) and preferably specific to everyday functional tasks.Progress to dynamic/mobility and rapid stepping exercises and introduce secondary motor or cognitive tasks to improve dual task performance.
**Sets/Repetitions**	≥8 exercises targeting muscles attached too or crossing the hip and spine At least 2 sets 8- 12 repetitions1- 3 min rest between sets	50-100 jumps per session divided into 3-5 sets of 10-20 repetitions. 1-2 min rest between sets.	Incorporate into daily activities or combine with resistance or impact exercise (e.g., balance for 10-30 s while waiting for kettle to boil, cooking or watching TV).
**Precautions**	Emphasize exercises performed in a standing (weight-bearing) position.Use caution with lifting weights higher than shoulder height to limit rotator cuff injury.For individuals with low spine BMD avoid spine flexion or twisting and encourage spine-sparing strategies.Include core stability and postural strengthening/endurance exercises as well as pelvic floor activities.	Teach correct landing technique.Progress slowly.Intersperse between strength and balance exercises.For those with incontinence issues first strengthen pelvic floor muscles and avoid jumping exercises with feet wide apart. For those with (osteo)arthritis, prescribe within limits of pain.	For individuals with impaired balance or high fracture risk, start with static and progress to dynamic balance exercises.

BW, body weight; RPE, Rating of Perceived Exertion; 1-RM, one-repetition maximum.

In accordance with most national physical activity guidelines, women should accumulate ≥150 min per week of moderate to vigorous intensity physical activity. To realistically accomplish all of the above therapeutic goals, one could combine activities e.g., lunges as a leg strengthening exercise that also challenges balance, step class that includes impact exercise and moderate/vigorous aerobic challenge and simultaneously challenges balance (91).

Exercising regularly has a beneficial effect on health but not every type of activity shows equal osteogenic effect. Previous studies about aerobic training such as swimming, cycling or walking and its positive impact on all body systems are contrary to those suggesting that these activities do not provide notable stimulus to bone and next to that do not cause an osteogenic effect. However there are types of activities which have positive influence on bone health. A lot of bone adaptive responses depends on magnitude, rate and frequency of loading. They must be dynamic, cyclic and induce relatively high bone strains. In order to elicit a bone system adaptive response relatively few loading cycles with adequate load intensity are required. Moreover breaks between repetition are equally or even more important than number of repetitions in cycle. Finally, loading diversification is required to stimulate an adaptive skeletal response (83).

## Summary

Based on the foregoing considerations, sclerostin is a marker to determine the effect of exercise on bone tissue processes. By inhibiting tissue formation processes, this protein mediates bone remodeling. In recent years, numerous studies have shown that properly selected physical training has a preventive effect on skeletal diseases, especially osteoporosis, by increasing bone mineral density (82, 84). This disease, which is considered to be a civilization disease, is a huge problem both socially and economically, so the fact of the beneficial effect of physical exercise as the cheapest and most beneficial cure is all the more convincing. This study demonstrates the relationship between the physical activity level and serum sclerostin level and bone mineral density, as osteogenic factors. This raises the question: why do near-maximal mechanical stress and high bone mineral density in athletes not correlate with reduced blood sclerostin levels, as in people with low or moderate activity levels? Are there other mechanisms involved in the osteogenic response with very high mechanical loading? Furthermore, it has been noted that not every type of physical activity results in a significant increase in BMD. According to selected studies osteogenic activity is affected by the load of exercise, type of physical training, and its effectiveness depends on the intensity and frequency of exercise, and the intervals between repetitions, among other factors. Moreover the very essential factors are gender and season, because in bone turnover markers secretion the seasonal variations was observed (85). The question remains, will osteoporosis be preventable and treatable in the near future with well-timed physical training as an alternative to medication?

There is still a need for further research to answer this question and to clearly establish the dynamics of sclerostin changes in relation to the factors influencing its secretion.

## Author contributions

Conceptualization: EM-C, AO, and AK. Writing—original draft preparation: AO, AK, MK, MC, EA, AS, FC and EM-C. Supervision: EM-C and FC. Project administration: EM-C. Funding acquisition: EM-C and AK. All authors have contributed to the article and approved the submitted version.

## Funding

This work was funded only by an internal grant from the Wroclaw University of Health and Sport Sciences. Project No. 503/62/05 “*Effectiveness of various therapeutic forms and their influence on nervous, muscular and vascular plasticity in patients after ischemic stroke*”.

## Conflict of interest

The authors declare that the research was conducted in the absence of any commercial or financial relationships that could be construed as a potential conflict of interest.

## Publisher’s note

All claims expressed in this article are solely those of the authors and do not necessarily represent those of their affiliated organizations, or those of the publisher, the editors and the reviewers. Any product that may be evaluated in this article, or claim that may be made by its manufacturer, is not guaranteed or endorsed by the publisher.

## References

[1] Sewerynek E Bajon K Stuss M . Secondary osteoporosis during long-term steroid treatment. Menopasal Rev (2007) 6:336–43.

[2] Khosala S Westendor JJ Oursler MJ . Building bone to reverse osteoporosis and repair fractures. J Clin Invest. (2008) 118(2):421–8. doi: 10.1172/JCI33612 PMC2214701 18246192

[3] Tong X Chen X Zhang S Huang M Shen X Xu J . The effect of exercise on the prevention of osteoporosis and bone angiogenesis. BioMed Res Int (2019) 2019:8171897. doi: 10.1155/2019/8171897 31139653 PMC6500645

[4] Föger-Samwald U Dovjak P Azizi-Semrad U Kerschan-Schindl K Pietschmann P . Osteoporosis: Pathophysiology and therapeutic options. EXCLI J (2020) 19:1017–37. doi: 10.17179/excli2020-2591 PMC7415937 32788914

[5] Czarkowska – Pączek B Wesołowska K Przybylski J . Exercise in prophylaxis of osteoporosis przy. Lek (2011) 68(2):103–6. 21751519

[6] Cosman F de Beur SJ LeBoff MS Lewiecki EM Tanner B Randall S . Clinician's guide to prevention and treatment of osteoporosis. Osteoporos Int (2014) 25(10):2359–81. doi: 10.1007/s00198-014-2794-2 PMC4176573 25182228

[7] Hernlund E Svedbom A Ivergard M Compston J Cooper C Stenmark J . Osteoporosis in the European union: medical management, epidemiology and economic burden. a report prepared in collaboration with the international osteoporosis foundation (IOF) and the European federation of pharmaceutical industry associations (EFPIA). Arch Osteoporos (2013) 8(1):1–115. doi: 10.1007/s11657-013-0136-1 PMC3880487 24113837

[8] Compston J . Osteoporosis: social and economic impact. Radiol Clin North Am (2010) 48(3):477–82. doi: 10.1016/j.rcl.2010.02.010 20609886

[9] Kucharska E . Osteoporosis: a social problem in the elderly population. Horizons of Education (2017) 16(40):37–57. doi: 10.17399/HW.2017.164003

[10] Horizons Educ . (2017) 16(40):37 57. doi: 10.17399/HW.2017.164003

[11] Kanis JA Norton N Harvey NC Jacobson T Johansson H Lorentzon M . SCOPE 2021: a new scorecard for osteoporosis in Europe. Arch Osteoporos (2021) 16(1):82. doi: 10.1007/s11657-020-00871-9 34080059 PMC8172408

[12] Abdolalipour S Mirghafourvand M Ghassab-Abdollahi N Farshbaf-Khalili A . Health-promoting lifestyle and quality of life in affected and unaffected menopausal women by primary osteoporosis. J Educ Health Promot (2021) 10:45. doi: 10.4103/jehp.jehp_450_20 34084792 PMC8057161

[13] Choi MH Yang JH Seo JS Y-j K Kang S-W . Prevalence and diagnosis experience of osteoporosis in postmenopausal women over 50: Focusing on socioeconomic factors. PloS One (2021) 16(3):e0248020. doi: 10.1371/journal.pone.0248020 33651848 PMC7924764

[14] Willers C Norton N Harvey NC Jacobson T Johansson H Lorentzon M . Osteoporosis in Europe: a compendium of country-specific reports. Arch Osteoporos (2022) 17:23. doi: 10.1007/s11657-021-00969-8 35079919 PMC8789736

[15] Dardzińska J Chabaj-Kędroń H Małgorzewicz S . Osteoporosis as a social disease : prevention methods. Hygeia Public Health (2016) 51(1):23–30.

[16] Ageing Europe - statistics on population developments. Luxembourg: Publications Office of the European Union, (2020). Available at: https://ec.europa.eu/eurostat/statistics-explained/index.php?title=Ageing_Europe_-_statistics_on_population_developments

[17] Janiszewska M Kulik T Dziedzic M Żołnierczuk-Kieliszek D Barańska A . Osteoporosis as a social problem – pathogenesis, symptoms and risk factors of postmenopausal osteoporosis. Probl Hig Epidemiol (2015) 96(1):106–14.

[18] Todd JA Robinson RJ . Osteoporosis and exercise. Postgrad Med J (2003) 79(932):320–3. doi: 10.1136/pmj.79.932.320 PMC1742726 12840119

[19] Malhan D Muelke M Rosch S Schaefer AB Merboth F Weisweiler D . An optimized approach to perform bone histomorphometry. Front Endocrinol (2018) 9:666. doi: 10.3389/fendo.2018.00666 PMC6259258 30519215

[20] Wilson N Hurkmans E Adams J Bakkers M Balážová P Baxter M . Prevention and management of osteoporotic fractures by non-physician health professionals: a systematic literature review to inform EULAR points to consider. RMD Open (2020) 6:e001143. doi: 10.1136/rmdopen-2019-001143 32144136 PMC7059534

[21] Perry SP Downey PA . Fracture risk and prevention: A multidimensional approach. Physic Ther (2012) 92(1):164–78. doi: 10.2522/ptj.20100383 21921251

[22] Cariati I Bonanni R Onorato F Mastrogregori A Rossi D Iundusi R . Role of physical activity in bone–muscle crosstalk: Biological aspects and clinical implications. J Funct Morphol Kinesiol. (2021) 6(2):55. doi: 10.3390/jfmk6020055 34205747 PMC8293201

[23] Moreira LD Oliveira ML Lirani-Galvão AP Marin-Mio RV Santos RN Lazaretti-Castro M . Physical exercise and osteoporosis: effects of different types of exercises on bone and physical function of postmenopausal women. Arq Bras Endocrinol Metabol. (2014) 58(5):514–22. doi: 10.1590/0004-2730000003374 25166042

[24] Troy KL Mancuso ME Butler TA Johnson JE . Exercise early and often: Effects of physical cctivity and exercise on women's bone health. Int J Environ Res Public Health (2018) 15(5):878. doi: 10.3390/ijerph15050878 29710770 PMC5981917

[25] Morris HA . Osteoporosis prevention–a worthy and achievable strategy. Nutrients. (2010) 2(10):1073–4. doi: 10.3390/nu2101073 PMC3257616 22253997

[26] Balemans W Ebeling M Patel N Van Hul E Olson P Dioszegi M . Increased bone density in sclerosteosis is due to the deficiency of a novel secreted protein (SOST). Hum Mol Genet (2001) 10:537–43. doi: 10.1093/hmg/10.5.537 11181578

[27] Winkler DG Yu C Geoghegan JC Ojala EW Skonier JE Shpektor D . Noggin and sclerostin bone morphogenetic protein antagonists form a mutually inhibitory complex. J Biol Chem (2004) 279:36293–8. doi: 10.1074/jbc.M400521200 15199066

[28] Delgado-Calle J Sato AY Bellido T . Role and mechanism of action of sclerostin in bone. J Bone (2017) 96:29–37. doi: 10.1016/j.bone.2016.10.007 PMC5328835 27742498

[29] Weivoda MM Youssef SJ Jo Oursler M . Sclerostin expression and functions beyond the osteocyte. J Bone (2017) 96:45–50. doi: 10.1016/j.bone.2016.11.024 PMC5328839 27888056

[30] Silverman SL . Sclerostin. J Osteoporos (2010) 2010:941419. doi: 10.4061/2010/941419 20981340 PMC2957275

[31] Pietrzyk B Smertka M Chudek J . Sclerostin: Intracellular mechanisms of action and its role in the pathogenesis of skeletal and vascular disorders. Adv Clin Exp Med (2017) 26(8):1283–91. doi: 10.17219/acem/68739 29264888

[32] Catalano A Bellone F Morabito N Corica F . Sclerostin and vascular pathophysiology. IJMS (2020) 21:4779. doi: 10.3390/ijms21134779 32640551 PMC7370046

[33] Yavropoulou MP Xygonakis C Lolou M Karadimou F Yovos JG . The sclerostin story: From human genetics to the development of novel anabolic treatment for osteoporosis. Hormones (2014) 13(4):476–87. doi: 10.14310/horm.2002.1552 25555179

[34] Sharma-Ghimire P Chen Z Sherk V Bemben D . Sclerostin and parathyroid hormone responses to acute whole-body vibration and resistance exercise in young women. J Bone Miner Metab (2019) 37:358–67. doi: 10.1007/s00774-018-0933-0 29956019

[35] Ardawi MS Rouzi AA Qari HM . Physical activity in relation to serum sclerostin, insulin-like growth factor-1 and bone turnover markers in healthy premenopausal women: a cross-sectional and a longitudinal study. J Clin Endocrinol Metab (2012) 97(10):3691–9. doi: 10.1210/jc.2011-3361 22865898

[36] Moester MJC Papapoulos SE Lowik CWGM van Bezooijen RL . Sclerostin: current knowledge and future perspectives. Calcif Tissue Int (2010) 87:99–107. doi: 10.1007/s00223-010-9372-1 20473488 PMC2903685

[37] Bonnet N Garnero P Ferrari S . Periostin action in bone. Mol Cell Endocrinol (2016) 432:75–82. doi: 10.1016/j.mce.2015.12.014 26721738

[38] Kramer I Halleux C Keller H Pegurri M Gooi JH Brander Weber P . Osteocyte wnt/beta-catenin signaling is required for normal bone homeostasis. Mol Cell Biol (2010) 30(12):3071–85. doi: 10.1128/MCB.01428-09 PMC2876685 20404086

[39] Bonnet N Standley KN Bianchi EN Stadelmann V Foti M Conway SJ . The matricellular protein periostin is required for sost inhibition and the anabolic response to mechanical loading and physical activity. J Biol Chem (2009) 284(51):35939–50. doi: 10.1074/jbc.M109.060335 PMC2791022 19837663

[40] Wolski H Drwęska – Matelska N Seremak – Mrozikiewicz A Łowicki Z Czerny B . The role of wnt/β-catenin pathway and LRP5 protein in metabolism of bone tissue and osteoporosis etiology. Ginekol Pol (2015) 86:311–4. doi: 10.17772/gp/2079 26117992

[41] Holdsworth G Roberts SJ Ke HZ . Novel actions of sclerostin on bone. J Mol Endocrinol (2019) 62(2):R167–85. doi: 10.1530/JME-18-0176 30532996

[42] Robling AG Niziolek PJ Baldridge LA Condon KW Allen MR Alam I . Mechanical stimulation of bone *in vivo* reduces osteocyte expression of sost/sclerostin. J Biol Chem (2008) 283:5866–75. doi: 10.1074/jbc.M705092200 18089564

[43] Lin C Jiang X Dai Z Guo X Weng T Wang J . Sclerostin mediates bone response to mechanical unloading through antagonizing wnt/β-catenin signaling. J Bone Miner Res (2009) 24:1651–61. doi: 10.1359/jbmr.090411 19419300

[44] Sharifi M Ereifej L Lewiecki EM . Sclerostin and skeletal health. Rev Endocr Metab Disord (2015) 16:149–56. doi: 10.1007/s11154-015-9311-6 25669441

[45] Cosman F Crittenden DB Adachi JD Binkley N Czerwinski E Ferrari S . Romosozumab treatment in postmenopausal women with osteoporosis. N Engl J Med (2016) 375(16):1532–43. doi: 10.1056/NEJMoa1607948 27641143

[46] Brandenburg VM D’Haese P Deck A Mekahli D Meijers B Neven E . From skeletal to cardiovascular disease in 12 steps–the evolution of sclerostin as a major player in CKD-MBD. Pediatr Nephrol (2016) 31:195–206. doi: 10.1007/s00467-015-3069-7 25735207

[47] Frings-Meuthen P Boehme G Liphardt AM Baecker N Hee M Rittweger J . Sclerostin and DKK1 levels during 14 and 21 days of bed rest in healthy young men. J Musculoskelet Neuronal Interact (2013) 13(1):45–52. 23445914

[48] Janik M Stuss M Michalska-Kasiczak M Jegier A Sewerynek E . Effects of physical activity on sclerostin concentrations. Endokrynol Pol (2018) 69(2):142–9. doi: 10.5603/EP.a2018.0008 29465155

[49] Bimonte VM Fittipaldi S Marocco C Emerenziani GP Fornari R Guidetti L . Physical activity and hypocaloric diet recovers osteoblasts homeostasis in women affected by abdominal obesity. Endocrine (2017) 58(2):340–8. doi: 10.1007/s12020-016-1193-1 27981516

[50] Wannenes F Papa V Greco EA Fornari R Marocco C Baldari C . Abdominal fat and sarcopenia in women significantly alter osteoblasts homeostasis *in vitro* by a WNT/β-catenin dependent mechanism. Int J Endocrinol (2014) 2014:278316. doi: 10.1155/2014/278316 24963291 PMC4054618

[51] Hinton PS Nigh P . Thyfault JSerum sclerostin decreases following 12 months of resistance- or jump-training in men with low bone mass. Bone (2017) 96:85–90. doi: 10.1016/j.bone.2016.10.011 27744012 PMC5328803

[52] Pickering ME Simon M Sornay-Rendu E Chikh K Carlier MC Raby AL . Serum sclerostin increases after acute physical activity. Calcif Tissue Int (2017) 101(2):170–3. doi: 10.1007/s00223-017-0272-5 28374174

[53] Gombos CG Bajsz V Pek E Schmidt B Sió E Molics B . Direct effects of physical training on markers of bone metabolism and serum sclerostin concentrations in older adults with low bone mass. BMC Musculoskelet Dis (2016) 17:254. doi: 10.1186/s12891-016-1109-5 PMC4899888 27278385

[54] Kouvelioti R Kurgan N Falk B Ward WE Josse AR Klentrou P . Response of sclerostin and bone turnover markers to high intensity interval exercise in young woman: does impact matter? BioMed Res Int (2018) 2018:4864952. doi: 10.1155/2018/4864952 30515401 PMC6236652

[55] Armamento-Villareal R Sadler C Napoli N Shah K Chode S Sinacore DR . Weight loss in obese older adults increases serum sclerostin and impairs hip geometry but both are prevented by exercise traning. J Bone Miner Res (2012) 27(5):1215–21. doi: 10.1002/jbmr.1560 PMC3361603 22392834

[56] Śliwicka E Cisoń T Pilczyńska-Szcześniak Ł Ziemba A Straburzyńska-Lupa A . Effects of marathon race on selected myokines and sclerostin in middle-aged male amateur runners. Sci Rep (2021) 11:2813. doi: 10.1038/s41598-021-82288-z 33531538 PMC7854637

[57] Zagrodna A Jóźków P Mędraś M Słowińska – Lisowska M . Sclerostin as a novel marker of bone turnover in athletes. Biol Sport (2016) 33:83–7. doi: 10.5604/20831862.1194125 PMC4763547 26929475

[58] Kouvelioti R Kurgan N Falk B WE W Josse AR Klentrou P . Response of sclerostin and bone turnover markers to high intensity interval exercise in young women: Does impact matter? BioMed Res Int (2018) 2018:1–8. doi: 10.1155/2018/4864952 PMC6236652 30515401

[59] Jürimäe J Tillmann V Cicchella A Stefanelli C Võsoberg K Tamm AL . Jürimäe, T increased sclerostin and preadipocyte factor-1 levels in prepubertal rhythmic gymnasts: associations with bone mineral density, body composition, and adipocytokine values. Osteoporos Int (2016) 27(3):1239–43. doi: 10.1007/s00198-015-3301-0 26323330

[60] Grasso D Corsetti R Lanteri P Di Bernardo C Colombini A Graziani R . Bone-muscle unit activity, salivary steroid hormones profile, and physical effort over a 3-week stage race. Scand J Med Sci Sports (2015) 25:70–80. doi: 10.1111/sms.12147 24433517

[61] Lombardi G Lanteri P Colombini A Mariotti M Banfi G . Sclerostin concentrations in athletes: role of load and gender. J Biol Regul Homeost Agents (2012) 26(1):157–63. 22475109

[62] Garnero P Sornay-Rendu E Munoz F Borel O Chapurlat RD . Association of serum sclerostin with bone mineral density, bone turnover, steroid and parathyroid hormones, and fracture risk in postmenopausal women: the OFELY study. Osteoporos Int (2013) 24:489–94. doi: 10.1007/s00198-012-1978-x 22525978

[63] Benedetti MG Furlini G Zati A Letizia Mauro G . The effectiveness of physical exercise on bone density in osteoporotic patients. BioMed Res Int (2018) 2018:4840531. doi: 10.1155/2018/4840531 30671455 PMC6323511

[64] Jürimäe J Karvelyte V Remmel L Al T Purge P Gruodyte-Raciene R . Sclerostin, preadipocyte factor 1 and bone mineral values in eumenorrheic adolescent athletes with diferent training patterns. J Bone Miner Metab (2020) 39(2):245–52. doi: 10.1007/s00774-020-01141-x 32880010

[65] Bonewald LF Johnson ML . Osteocytes, mechanosensing and wnt signaling. Bone (2008) 42:606. doi: 10.1016/j.bone.2007.12.224 18280232 PMC2349095

[66] Isaksson H Gröngröft I Wilson W van Donkelaar C van Rietbergen B Tami A . Remodeling of fracture callus in mice is consistent with mechanical loading and bone remodeling theory. J Orthop Res (2009) 27:664. doi: 10.1002/jor.20725 18985689

[67] Hong AR Kim SW . Effects of resistance exercise on bone health. Endocrinol Metab (Seoul) (2018) 33(4):435–44. doi: 10.3803/EnM.2018.33.4.435 PMC6279907 30513557

[68] Kemmler W Shojaa M Kohl M von Stengel S . Effects of different types of exercise on bone mineral density in postmenopausal women: A systematic review and meta-analysis. Calcif Tissue Int (2020) 107:409–39. doi: 10.1007/s00223-020-00744-w PMC7546993 32785775

[69] Stengel SV Kemmler W Pintag R Beeskow C Weineck J Lauber D . Power training is more effective than strength training for maintaining bone mineral density in postmenopausal women. J Appl Physiol (2005) 99:181. doi: 10.1152/japplphysiol.01260.2004 15746294

[70] Bailey CA Brooke-Wavell K . Optimum frequency of exercise for bone health: randomised controlled trial of a high-impact unilateral intervention. Bone (2010) 46:1043. doi: 10.1016/j.bone.2009.12.001 20004758

[71] Siller-Jackson AJ Burra S Gu S Xia X Bonewald LF Sprague E . Adaptation of connexin 43-hemichannel prostaglandin release to mechanical loading. J Biol Chem (2008) 283:26374. doi: 10.1074/jbc.M803136200 18676366 PMC2546557

[72] Borer KT Fogleman K Gross K La New JM Dengel D . Walking intensity for postmenopausal bone mineral preservation and accrual. Bone (2007) 41:713. doi: 10.1016/j.bone.2007.06.009 17686670

[73] Robling AG Hinant FM Burr DB Turner CH . Improved bone structure and strength after long-term mechanical loading is greatest if loading is separated into short bouts. J Bone Miner Res (2002) 17:1545. doi: 10.1359/jbmr.2002.17.8.1545 12162508

[74] Srinivasan S Ausk BJ Poliachik SL Warner SE Richardson TS . Gross TS rest- inserted loading rapidly amplifies the response of bone to small increases in strain and load cycle. J Appl Physiol (2007) 102:1945. doi: 10.1152/japplphysiol.00507.2006 17255366

[75] Gross TS Poliachik SL Ausk BJ Sanford DA Becker BA Srinivasan S . Why rest stimulates bone formation: a hypothesis based on complex adaptive phenomenon. Exerc Sport Sci Rev (2004) 32:9. doi: 10.1097/00003677-200401000-00003 14748543 PMC1435735

[76] Hejazi K Askari R Hofmeister M . Effects of physical exercise on bone mineral density in older postmenopausal women: a systematic review and meta-analysis of randomized controlled trials. Arch Osteoporos. (2022) 17(1):102. doi: 10.1007/s11657-022-01140-7 35896850

[77] Kohrt WM Ehsani AA Birge SJ Jr. Effect of exercise involving predominantly either joint-reaction or ground-reaction forces on bone mineral density in older women. J Bone Miner Res (1997) 12:1253. doi: 10.1359/jbmr.1997.12.8.1253 9258756

[78] Kemmler W Weineck J Kalender WA Engelke K . The effect of habitual physical activity, non-athletic exercise, muscle strength, and VO2max on bone mineral density is rather low in early postmenopausal women. J Musculosceletal Neuron Interact (2004) 4:325. 15615501

[79] Kirmani S Christen D van Lenthe GH Fischer PR Bouxsein ML McCready LK . Bone structure at the distal radius during adolescent growth. J Bone Miner Res (2009) 24(6):1033–42. doi: 10.1359/jbmr.081255 PMC2683647 19113916

[80] Warden SJ Fuchs RK Castillo AB Nelson IR Turner CH . Exercise when young provides lifelong benefits to bone structure and strength. J Bone Miner Res (2007) 22(2):251–9. doi: 10.1359/jbmr.061107 17129172

[81] Warden SJ Mantila Roosa SM Kersh ME Hurd AL Fleisig GS Pandy MG . Physical activity when young provides lifelong benefits to cortical bone size and strength in men. Proc Natl Acad Sci U.S.A. (2014) 111(14):5337–42. doi: 10.1073/pnas.1321605111 PMC3986122 24706816

[82] Amato A Baldassano S Cortis C Cooper J Proia P . Physical activity, nutrition, and bone health. Hum Mov (2018) 19(4):1–10. doi: 10.5114/hm.2018.77318

[83] Daly RM Dalla Via J Rachel L. Duckham RL Fraser SF Wulff Helge E . Exercise for the prevention of osteoporosis in postmenopausal women: an evidence-based guide to the optimal prescription. Braz J Phys Ther (2019) 23(2):170–80. doi: 10.1016/j.bjpt.2018.11.011 PMC6429007 30503353

[84] Beck BR Daly RM Fiatarone Singh MA Taaffe DR . Exercise and sports science Australia (ESSA) position statement onexercise prescription for the prevention and management ofosteoporosis. J Sci Med Sport (2017) 20(5):438–445. doi: 10.1016/j.jsams.2016.10.001 27840033

[85] Mero A Häkkinen K Kyröläinen H Mero A . Effects of training on bone metabolism in young athletes. Hum Mov (2021) 22(4):105–12. doi: 10.5114/hm.2021.104181

